# Effectiveness of a short-term oxygen therapy training program in Liberia during and after COVID-19

**DOI:** 10.3389/fpubh.2025.1490134

**Published:** 2025-02-12

**Authors:** Mark B. Luke, Moses Ziah, Lily Zhi Ning Lu, Michael D. Davis, Samson Arzoaquoi, Eva Drucker, Foday Kanneh, Gorbee G. Logan, Moses Massaquoi

**Affiliations:** ^1^Liberia Ministry of Health, Monrovia, Liberia; ^2^Liberia National COVID-19 Incidence Management System, Monrovia, Liberia; ^3^Clinton Health Access Initiative, Monrovia, Liberia; ^4^Wells Center for Pediatric Research/Pediatric Pulmonology, Allergy, and Sleep Medicine, Riley Hospital for Children at Indiana University School of Medicine, Bloomington, IN, United States; ^5^Partner Liberia, Monrovia, Liberia

**Keywords:** COVID-19, oxygen, respiratory care, in-service training, case management, pandemic response

## Abstract

**Background:**

Access to high-quality medical oxygen has been a long-standing challenge in Liberia due to barriers that span across the health system, which were amplified during the COVID-19 pandemic. The surge in cases requiring oxygen therapy necessitated rapid capacity-building for healthcare workers. In response, an emergency oxygen training package was adapted and implemented by the Liberia Ministry of Health and the National Incidence Management System. This manuscript evaluates the implementation of a short-term respiratory care training package to rapidly build healthcare worker capacity during the COVID-19 response and its adaptation for routine in-service training post-response.

**Methods:**

The emergency training used the “hot and cold” simulation approach from the 2014 Ebola response, consisting of a cold phase (3-days) with didactic lectures and practical sessions, and mock COVID treatment unit simulations (2-days); and a hot phase within an active CTU. Participants were doctors, physician assistants, nurses, or midwives, deployed to COVID treatment units at major health centers and hospitals across all counties in Liberia. Training assessments consisted of a paper-based knowledge test pre- and post-training, and Objective Structured Clinical Examinations post-training.

**Results:**

The emergency training as part of COVID response included 123 health care workers from 43 health facilities and saw a significant increase in knowledge (median score of 46% pre-training vs. 84% post-training, *p* < 0.001). Adaptation and piloting of the package for routine in-service training was also effective at increasing knowledge amongst 81 health care workers (median score of 41% pre-training vs. 78% post-training, *p* < 0.001). High post-training Objective Structured Clinical Examination scores demonstrated clinical competency achievement in both cohorts. For emergency training, median scores were 92% (pulse oximetry), 81% (oxygen cylinders), and 83% (oxygen concentrators). For routine in-service training, scores were 88, 82, and 84%, respectively.

**Conclusion:**

We demonstrate that the implementation of a healthcare worker training package in oxygen therapy during the COVID response in Liberia and its eventual integration into a routine in-service training program was able to achieve significant improvements in health care worker knowledge and skills. This highlights the feasibility of using rapid and short-term training to enhance clinical capacity within both emergency and post-response settings in a resource-limited country.

## 1 Background

Oxygen, recognized as an essential medicine, holds profound significance across various medical disciplines due to its critical role in sustaining life and improving health outcomes. From supporting vital functions in Reproductive, Maternal, Newborn, Child, and Adolescent Health (RMNCAH) to being a cornerstone in emergency and critical care, as well as in addressing Non-Communicable Diseases and Injuries (NCDIs), the indispensability of oxygen cannot be overstated. As emphasized in Liberia’s national oxygen roadmap (1), the availability and appropriate use of oxygen are paramount in ensuring the effective management of health conditions, underscoring its pivotal role in healthcare delivery and its profound impact on patient care and recovery.

Liberia, like many low- and middle-income countries (LMICs), has long faced challenges in delivering high-quality respiratory care and oxygen therapy due to gaps in the availability of medical oxygen technologies and infrastructure, and a shortage of healthcare workers (HCW) trained on oxygen therapy (2, 3). National efforts to improve access to medical oxygen have been underway for the past decade, including initiatives to improve healthcare workers’ knowledge and skills to deliver oxygen therapy. Efforts to enhance capacity have primarily concentrated on providing in-service training, as oxygen therapy was not typically included in pre-service training programs for healthcare professionals (physicians receive some exposure during pre-service training). In-service training programs have also been predominantly focused on neonatal resuscitation, exemplified by initiatives like Helping Babies Breathe (4–6).

To introduce specialized training in respiratory therapy, the Liberia Respiratory Care Institute (LRCI) was established in 2012, utilizing a curriculum based on the United States (U.S.) National Board of Respiratory Care and guidelines of the American Association for Respiratory Care. LRCI provides an associate degree and graduates were board-eligible for the Respiratory Therapist license accredited by the Liberia Medical and Dental Council (LMDC) in 2015. In 2018, the Liberia Ministry of Health (MOH) also established the respiratory care department under its Emergency Medical Response (EMR) Unit.

When Liberia recorded its first case of COVID-19 in March 2020, existing challenges associated with oxygen therapy were amplified. A surge of moderate and severe cases requiring medical oxygen in the ensuing months posed an urgent need to rapidly increase HCW capacity, all within the context of lacking nationally standardized practice guidelines or training packages for oxygen therapy. An assessment was undertaken by the Liberia MOH with support from the Clinton Health Access Initiative (CHAI) between September to November 2020 to determine the then availability of oxygen technologies and readiness of major health facilities in Liberia to provide medical oxygen (7). Data were collected from a total of 53 secondary and tertiary health centers and hospitals, from the public and private sector. As oxygen therapy was not included in the pre-service curriculum of the majority of cadres, a proxy was used to determine availability of HCWs trained on oxygen therapy in the various facilities by evaluating whether a facility had either a medical doctor or a nurse anesthetist. 74% of facilities had at least one medical doctor, and 62% of facilities had at least one nurse anesthetist; however, the provider-patient ratio was 0.2 doctors per 1,000 population. An updated assessment in 2023 covering 57 health facilities found that between 2019 and 2023, a little over half (67%) of secondary and tertiary health facilities reported having staff trained on oxygen therapy (8). This indicates a significant gap in availability HCWs to provide oxygen therapy, but also presents an opportunity to support task-shifting of basic oxygen therapy to other mid-level cadres.

To add to documented evidence on practical approaches to increase HCW knowledge and skills to deliver oxygen therapy, the MOH, supported by CHAI, Partner Liberia, and others evaluated the effectiveness of a short-term respiratory care training package developed for mid-level HCW cadres in Liberia as part of the government’s national COVID-19 response coordinated through the Liberia National Incidence Management System (IMS). This paper presents learnings and outcomes from the implementation of HCW training through rapid scale-up as a response to COVID-19, which was then integrated into a routine in-service training program for oxygen therapy in Liberia. Drawing upon field experiences in Liberia, this paper aims to synthesize real-world experiences and lessons learned that will be valuable for the adaptation of effective in-service training strategies in other resource-constrained settings.

## 2 Methodology

The study employed a prospective pre-post intervention design to evaluate the effectiveness of oxygen therapy training. Participants’ knowledge and skills were assessed before and after the training intervention, allowing for paired comparisons of the same group at two time points. Paper-based knowledge pre-tests were administered at the beginning of the training, while post-tests and Objective Structured Clinical Examinations (OSCEs) were conducted at the end. The selection of trainees for both the emergency and routine training programs was based on programmatic criteria rather than aiming for the representativeness of healthcare cadres in Liberia. The number of participants was determined by the specific programmatic needs of the COVID-19 response and the subsequent routine in-service training initiative, rather than by statistical power calculations for hypothesis testing. This approach ensured that the training targeted healthcare workers to bridge the human resources gap during and post-COVID response.

The training were led by a diverse team comprising MOH staff, such as respiratory therapists and clinicians from the national IMS, along with clinical personnel from health sector implementation partners. The assessments were administered and scored by the training facilitators. These facilitators bring extensive experience in clinical oxygen therapy and a strong background in conducting healthcare training.

To ensure quality control in data collection and entry, standardized scoring rubric was used for both the paper-based tests and the OSCEs. Double-checking of scores was performed by a second facilitator to minimize errors. Data entry was conducted by designated team members using a standardized Excel template, with random spot-checks performed by a supervisor to verify accuracy. Any discrepancies identified during these quality control measures were resolved through discussion among the facilitators and, if necessary, by reviewing the original assessment documents. Although the number of questions in the knowledge test varied between emergency and in-service training, participants’ scores were calculated as the percentage of correct answers.

The emergency training and in-service training differed in several key aspects. The emergency training curriculum featured more focused modules on COVID-19 and related infection prevention and control (IPC) measures, as well as oxygen administration algorithms specifically tailored for COVID-19 case management. In contrast, the in-service training content took a broader approach, focusing on oxygen use in non-COVID response settings. The emergency training also incorporated additional “hot” training sessions conducted within COVID treatment units (CTUs), lasting 1–2 days. Furthermore, the emergency training targeted healthcare providers working directly in CTUs, whereas the in-service training was designed for a wider range of facility providers. These differences reflect the specialized nature of the emergency training in addressing the immediate needs of the COVID-19 response.

### 2.1 Emergency oxygen training for COVID-19

As part of the COVID-19 response to rapidly increase the knowledge and skills of HCW working in COVID-19 treatment units (CTU), an emergency oxygen training package was rapidly developed based on a draft in-service training package. The training package covered a range of topics to be delivered over the course of 6 days. The training utilized a similar approach to rapid capacity-building conducted during Liberia’s 2014 Ebola outbreak, consisting of “cold” and “hot” training phases (Table 1). The first 3 days of the training consisted of didactic lectures and presentations, demonstrations, and practical sessions. Days four and five of the training consisted of clinical simulations within a mock CTU setting (“cold”). Day six consisted of shadowing in an active CTU (“hot training”) that could be accessed, with a preference for the Star Base CTU located in Montserrado County, which was the facility with the highest response level, when logistically possible. From there, clinicians were sent back to their assigned facilities and ongoing mentoring by central MOH or IMS facilitators was conducted as necessary.

**Table 1:** Syllabus for emergency oxygen training for COVID-19.

Approach	Training day	Modules and topics
Cold training	Day 1	Introduction to COVID-19 Hypoxemia & indications for oxygen therapy Use of pulse oximetry Oxygen sources Oxygen regulation & conditioning Oxygen delivery interface
	Day 2	Administering oxygen using cylinders and concentrators
	Day 3	Infection prevention and control (IPC) standard precautions and transmission-based precautions Personal protective equipment (PPE) donning and doffing
	Day 4	Clinical simulations in mock CTUs and extensive skills stations
	Day 5	
Hot training	Day 6	CTU orientation and shadowing at Star Base CTU or equivalent

Staff from a range of clinical cadres who were assigned to CTUs were enrolled to participate in the emergency training across all 15 counties in Liberia. The training was facilitated by a mixture of MOH staff, such as respiratory therapists from the EMR Unit; clinicians from the national IMS Case Management Pillar; and clinical staff from health sector implementation partners.

During the emergency training, the competency assessments included a paper-based test as well as Objective Structured Clinical Examinations (OSCE) to evaluate practical hands-on skills. The paper-based test consisted of 13 questions on oxygen therapy indications, administration, and safety. The questions were administered to training participants prior to the first training session without being provided any study materials, and the same examination was administered at the end of all training sessions (on day 3 or 4). During practical sessions throughout the training, clinical checklists were used to guide participants through hands-on practice. These checklists were adapted to also serve as the rubric for the administration of OSCEs to assess post-training competency in the practical use and application of key oxygen technologies, including pulse oximeters, oxygen concentrators, and oxygen cylinders. OSCEs were administered on day 3 and 4 of the training.

### 2.2 National in-service training on basic medical oxygen therapy

To ensure that the government and its partners are prepared to continue high-quality clinical capacity-building efforts beyond the COVID response, as part of the country’s longer-term efforts to scale up respiratory care capacity, the emergency oxygen training package was adapted into a more comprehensive national in-service training package for basic oxygen therapy. The training package was validated by the Liberia MOH and its oxygen partners through the national oxygen technical working group to standardize in-service training for clinicians to diagnose hypoxemia and administer basic oxygen therapy. While the training package focuses on oxygen therapy, it is expected that the modular format of the package will allow it to be adapted as part of any broader, integrated in-service training package (for example, integration into in-service RMNCAH training). To complement the training package, the National Clinical Guidelines for Hypoxemia Management and Oxygen Therapy were also validated in June 2022.

Duration of the in-service training is intended to be flexible, depending on baseline knowledge, resource availability, and the need for any module-specific deep-dives, but it is recommended to be between 5 and 10 days. During the implementation described in this paper, training was conducted over the course of 5 days for three cohorts, consisting of county and district-level clinical supervisors (“subnational mentors”) who are MOH employees in one cohort, and facility-based providers over two cohorts. The training was facilitated by respiratory therapists from the MOH EMR Unit, supported by practicing physicians and clinical staff from non-government partners.

Competency assessments also utilized a combination of a paper-based test (expanded to 27 questions) administered pre-training and post-training, plus post-training OSCEs focused on pulse oximeters, oxygen concentrators, and oxygen cylinders.

### 2.3 Statistical analysis

For both the Emergency Oxygen Training for COVID-19 and the National In-Service Training on Basic Medical Oxygen Therapy, participants completed multiple-choice examinations before and after the training. The data were analyzed using Excel and SigmaStat (SyStat Software, Inc., San Jose, CA, USA), with descriptive analyses undertaken to generate frequencies, percentages, and means.

To evaluate the difference in average participant pre-training and post-training examination scores, paired t-tests were used for normally distributed data. For non-Gaussian distributed data, as determined by the Shapiro–Wilk test, the Wilcoxon rank-sum test was applied. These statistical analyses allow for a quantitative assessment of the training’s effectiveness in improving participants’ knowledge and understanding of advanced respiratory care for COVID-19 patients and basic medical oxygen therapy.

The findings were visualized through a variety of charts and figures to facilitate a deeper understanding of the results, with analysis disaggregated between the emergency training dataset and the routine in-service training dataset. A bar chart displayed the breakdown of the datasets by county, cadre, and the number of facilities in each county, while box plots showed the minimum, maximum, range, mean, and median values for pre-test and post-test results.

## 3 Results

### 3.1 Emergency oxygen training for COVID-19

**Figure 1:**
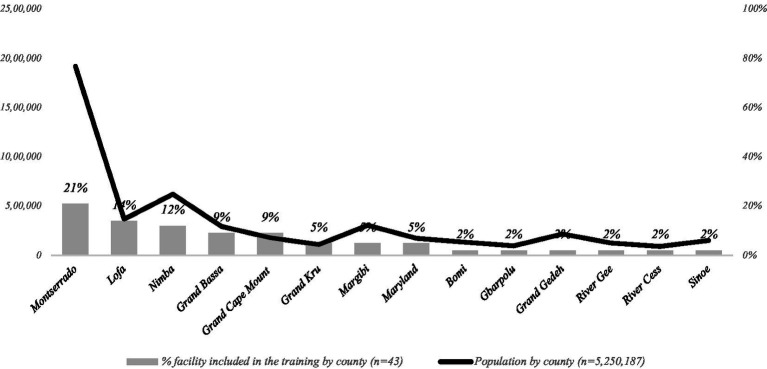
Health facilities covered by COVID emergency oxygen training, by county with population distribution.

A total of 123 HCW from all 15 counties in Liberia took part in the training program (Figure 1). These HCWs were spread across 43 health centers (*n* = 10, 23%) and hospitals (*n* = 33, 77%), classified as secondary or tertiary health facilities, encompassing both public (*n* = 32, 74%,) and private (*n* = 11, 26%) sectors. The training sessions were divided into seven regional cohorts, uniting participants from healthcare facilities in neighboring counties for collaborative learning. These trainings occurred between July and November 2021.

The training cohorts consisted of professionals from five healthcare cadres: midwives (7%), nurses (66%), nurse anesthetists (8%), physician assistants (11%), and physicians (7%). Two nursing trainees were also included.

#### 3.1.1 Pre-training knowledge assessment

**Figure 2:**
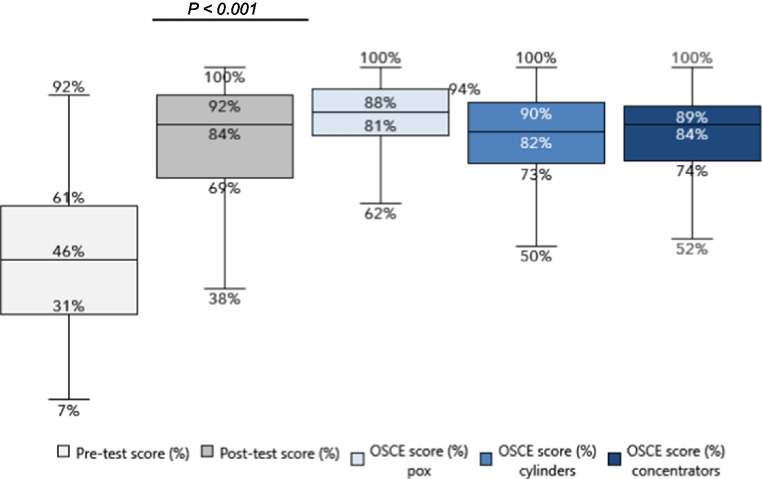
COVID-19 emergency oxygen training pre vs. post-test results and OSCE results.

Pre-training scores with regards to baseline knowledge of basic oxygen therapy ranged from a minimum score of 7% to a maximum of 92% (IQR = 30) (Figure 2). The pre-test results show a median score of 46% and an average score of 46% with a standard deviation of 0.20, indicating that the initial performance levels are low, centered around 46% with a moderate level of variability. The results suggest a homogeneous starting point for the training program, providing a stable baseline for assessing the impact of the oxygen training intervention on performance outcomes.

#### 3.1.2 Post-training knowledge assessment

There was a significant improvement in the range of scores post-training, with a minimum score of 38% to a maximum of 100% (IQR = 23), and a median score of 84%. The post-training average score was 80% (*p* < 0.001) with a standard deviation of 0.16, demonstrating significant improvements from the pre-training baseline and with reduced variability in score.

While the emergency training package was primarily designed to target mid-level cadres, some doctors participated in the training at the explicit request of the facilities during the height of the COVID-19 response. A comparative analysis of the COVID-19 emergency oxygen training test scores by cadre reveals that doctors had the best performance in both pre- and post-training assessments, with physician assistants (PAs) closely following. Nurse anesthetists had higher pre-training performance than PAs, but a lower post-training average. The results also demonstrate performance differences between doctors and nurses, as well as between doctors and midwives. When statistical analyses were conducted per group, it was found that all groups showed statistically significant improvement: *p* < 0.001 except doctors (*p* < 0.007) and nurse anesthetists (*p* < 0.005).

#### 3.1.3 Post-training skills assessment (OSCE)

The post-training OSCE evaluated the participants’ practical skills in the use of the pulse oximeter (for hypoxemia diagnosis), and use of the oxygen cylinder and the oxygen concentrator for oxygen delivery (participants also demonstrated the use of appropriate consumables such as simple face masks, non-rebreather masks, and of accessories such as cylinder flowmeters and humidifier bottles). Participants were generally slightly more proficient in the use of the pulse oximeter compared to the other devices: the median score was observed to be 88% for pulse oximeter use (IQR = 13), 82% for cylinder use (IQR = 17), and 84% for concentrator use (IQR = 15).

### 3.2 National in-service training on basic oxygen therapy

**Figure 3:**
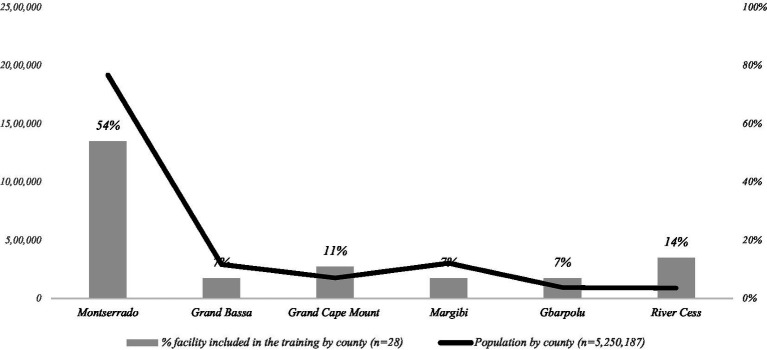
Health facilities covered by routine in-service oxygen training, by county with population distribution.

To leverage positive spill-over effects on maternal and child health, the initial use of the national in-service training package was concentrated in four counties in Liberia where CHAI has been supporting MOH on RMNCAH capacity-building: Montserrado, Gbarpolu, Grand Bassa, and Rivercess. A few providers from Grand Cape Mount and Margibi counties were included at the Ministry’s request. A total of 81 HCW, distinct from those who attended the COVID emergency training, participated in the piloting of the routine in-service training on basic oxygen therapy. These participants were assigned across 28 public hospitals (36%), and health centers (50%), and also included subnational (i.e., county and district-level) supervisors/mentors from the Ministry’s county and district health teams (14%). Some health facilities whose HCWs participated in the emergency training sent additional providers for the in-service training, but otherwise, the primary purpose of the in-service training was to expand oxygen services to additional facilities (Figure 3). The training was conducted in three cohorts and included professionals from five cadres: midwives (40%), nurses (48%), nurse anesthetists (2%), physician assistants (7%), and nurse trainees (2%).

#### 3.2.1 Pre-training knowledge assessment

**Figure 4:**
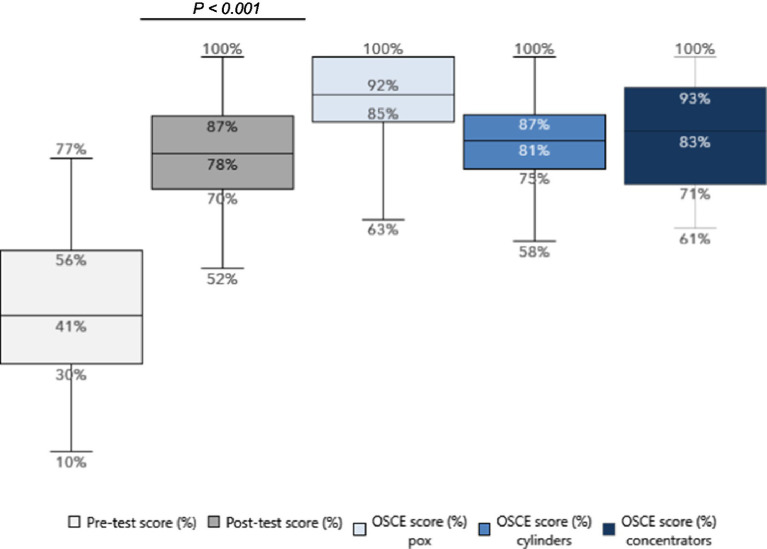
National routine in-service oxygen training pre vs. post-test results and OSCE results.

The pre-test scores for the routine in-service training ranged from a minimum score of 10% to a maximum of 77% (IQR = 26), with a median score of 41% (Figure 4). The average score across all participants was 43% with a standard deviation of 0.16.

#### 3.2.2 Post-training knowledge assessment

There was a significant improvement in the range of scores post-training, with a minimum score of 52% and a maximum of 100% (IQR = 17), and a median score of 78%. Post-training average score was also 78% (*p* < 0.001) with a standard deviation of 0.11, demonstrating significant improvements from the pre-training baseline and with reduced variability in score. Among the participants, 60 were facility-based providers, and 21 were subnational mentors. Despite facility-based providers initially scoring lower on the pre-test compared to subnational mentors (42% vs. 49%), the post-test results did not vary significantly (76% vs. 75%). Further analysis of results by cadre revealed that nurses and midwives showed highly significant improvements (*p* < 0.001), while PAs also demonstrated significant progress (*p* = 0.002). The sample size for Nurse Anesthetists was insufficient to perform a meaningful statistical analysis for this particular subgroup.

#### 3.2.3 Post-training skills assessment (OSCE)

Similar to the emergency training, participants were also asked to demonstrate their practical skills in the use of key oxygen technologies as part of the post-training assessment. The median OSCE score was 92% for the use of pulse oximeters; 81% for the use of cylinders; and 83% for the use of concentrators.

## 4 Discussion

Oxygen training has not historically been integrated into pre-service training of clinicians in Liberia, and prior to 2021, the country had no national in-service training program for oxygen therapy. The quality of in-service training is often marred by the lack of standardization and the lack of essential equipment and supplies for hands-on practice. These gaps resulted in the low baseline test scores across all cohorts in this study. Performance differences were observed between clinical cadres who spent more time in pre-service training (e.g., physicians and PAs) compared to those with fewer years of study (e.g., nurses and midwives). However, the sample size did not allow for determination of whether the better pre-training knowledge of physicians and PAs was statistically significant. The assessment scores and feedback from participants across all cadres indicate the usefulness of the training contents, regardless of their prior training experience.

Despite limited knowledge in hypoxemia diagnosis and oxygen therapy observed in the participating HCW, a large portion of them were able to attain a post-training score exceeding the 70% certification threshold set by the MOH (93% of emergency training participants and 77% of routine training participants). There was reduced variability in post-test scores compared to pre-test scores, suggesting a homogeneous level of knowledge among the trainees after the training, even across different cadres of providers with varying pre-service qualifications.

Participants also demonstrated proficiency in the use of key oxygen technologies such as pulse oximeters, oxygen cylinders, and oxygen concentrators, as demonstrated through practical skills assessments post-training. Training planners, facilitators and participants attributed the achievements in participant competencies to demonstrations and practical sessions that formed part of the training, beyond didactic sessions on theory that often form the entirety of other in-service trainings. Facilitators and participants remarked on the benefits of allocating substantial time to gain hands-on experience on the proper use of devices and supplies, to demonstrate techniques on anatomical manakins, and to be tested with case scenarios for diagnosis and management. Future trainings in oxygen or other in-service programs should utilize a similar approach.

The comparable and consistent achievements observed across different cadres, different training cohorts, and locations suggest that the training approach used is feasible and scalable, and that the training contents are robust. The short-term oxygen training package described in this paper is thus highly scalable within the context of Liberia, and potentially to other similar settings where there are insufficient pre-service preparations or HCWs report low confidence in clinical practice (9). The training can be easily replicated to cover additional health facilities and HCWs: the training utilized local facilitators within the MOH and its affiliated institutions (the trainings were also an opportunity for external implementing partners to further build the capacity of local facilitators); all training materials including presentations, knowledge and skills assessments, case scenarios, and job aids have been developed and validated in its effectiveness; equipment and supplies procured for the pilot phase can be reused in future practical sessions. Furthermore, the inclusion of subnational mentors in the trainings described was a solution to improve sustainability of capacity-building, such that central and subnational MOH teams can conduct ongoing facility-based mentoring following cluster trainings. We recommend this follow-up mentoring approach which has been demonstrated to increase cost-effectiveness and sustainability when compared to one-off trainings (10–12).

These training efforts also had broader implications for the enabling environment for oxygen access in Liberia. Prior to these trainings, the prescription of oxygen therapy was limited to physicians; access is limited due to a severe shortage of physicians in the country, even at higher level facilities. Oxygen therapy was then either administered only by doctors, or by mid-level HCWs who have not been formally trained in the procedure. The emergency and in-service trainings described here have allowed for oxygen therapy to be task-shifted to additional cadres, which is essential to bridge the access gap in a country where the skilled birth attendant (SBA) to population ratio is significantly below the WHO recommendation of 2.28 per 1,000 population. With the task-shifting, mid-level providers, now trained on oxygen therapy including determination of flowrates and target SpO2, are able to consult and work together with physicians to determine the best course of management. Additionally, the Liberia MOH was able to develop national clinical guidelines to standardize hypoxemia diagnosis and management using oxygen therapy, marking the first time that global best practices in oxygen therapy have been adapted in the country to improve patient care. To further institutionalize oxygen therapy training across other disease areas, we also recommend that oxygen training modules be integrated into pre-service training for clinicians and integrated into other related in-service training (for example, as part of emergency obstetrics care, newborn care, and integrated management of childhood illness).

Our findings in Liberia align with those from studies on oxygen therapy from other resource-constrained environments, and further builds upon them with our unique approach. In Uganda, Dauncey et al. observed significant improvements in healthcare workers’ knowledge and skills following oxygen therapy training, with mean knowledge scores increasing from 29 to 57% post-training (13). While our results demonstrate higher post-training scores (84%), the relative improvement is comparable, suggesting the effectiveness of targeted training programs across different contexts. Similarly, in Nigeria, Bakare et al. implemented an emergency oxygen therapy training program that achieved a 40% increase in participants’ knowledge scores, consistent with our observations (14). This underscores the potential for knowledge transfer in resource-limited settings when appropriate training methodologies are used.

The “hot and cold” simulation approach utilized in Liberia appears particularly effective when compared to other methodologies. For instance, a study in Ethiopia by Hunie et al., which employed only classroom-based training, reported lower practical skill acquisition (OSCE scores of 72–78%) compared to our results (88–92%). This suggests that the combination of simulated and real clinical environments in our emergency trainings played a role to enhance skill retention and practical application.

### 4.1 Limitations

While this study demonstrates positive outcomes in knowledge and skills attainment amongst trained HCWs, several limitations should be acknowledged. First, due to resource and time constraints, the trainings did not conduct pre-training OSCEs to evaluate practical skills prior to the workshops. However, given the participants’ very low baseline knowledge scores, OSCE scores likely would have been considerably low. Our analyses of pre- vs. post-training assessments also face challenges in establishing causality, as demonstrating a direct causal relationship between the training program and the observed changes did not consider analyses with additional control variables. Furthermore, the knowledge and skills assessments do not consider critical aspects such as behavioral changes, practical application of learning in real-world scenarios, and the translation of skills into overall job performance and patient care quality.

The post-training assessments captured immediate changes in HCW knowledge and capacity. However, routine quantitative follow-ups are necessary to evaluate the long-term retention of training outcomes among participants. While some facility-based mentoring visits have been started to ascertain skills and knowledge retention (as well as build the capacity of additional providers who did not participate in the cluster trainings), there is a need to reinforce the use of clinical checklists for routine evaluation of provider competencies in a health facility setting, and to more rigorously document mentoring observations to gauge the enduring impact of training programs on healthcare providers’ competencies over time.

There has been a push by Liberia MOH to begin the routine capture of oxygen-related patient data in health facilities in Liberia, but implementation of high-quality data collection will take time; thus, despite qualitative feedback from MOH and facility providers, we are unable to quantitively correlate changes in patient outcomes and quality of care with the observed improvements in provider knowledge and skills. This underscores the importance of not only routine data, but also longitudinal research to assess the practical impact of HCW capacity-building initiatives. Finally, while the training package has demonstrated effectiveness within Liberia’s health system, additional investigations are needed to adapt the package for use across more diverse healthcare contexts.

## 5 Conclusion

We demonstrate that the implementation of an HCW training package in oxygen therapy during the COVID-19 response in Liberia, as well as its eventual integration into a routine in-service training program, was able to achieve significant improvements in HCW knowledge and skills, highlighting the effectiveness of rapid and short-term trainings to enhance clinical capacity in providers within both emergency and post-response settings. We demonstrate that the oxygen training packages tested here can be easily scaled to increase service coverage in similar contexts, suggesting that respiratory care delivery could potentially be improved in LMICs through focused, short-term training modules, even in HCWs without prior significant respiratory care training. We also emphasize the importance of a comprehensive training approach that is inclusive of theory and practical sessions, as well as recommend the use of ongoing facility-based mentoring for follow-up capacity-building.

This paper serves as a crucial contribution to the existing body of knowledge due to the scarcity of published literature and practical insights from sub-Saharan Africa regarding oxygen training for a diverse and multidisciplinary health workforce. The dissemination of practical implementation experience related to clinical capacity-building in resource-constrained settings can provide important insights on specific challenges and help formulate best practices to strengthen a key building block of the health system (15).

## Data Availability

The original contributions presented in the study are included in the article/supplementary material, further inquiries can be directed to the corresponding authors.

## References

[1] Liberia Ministry of Health. National roadmap to increase access to medical oxygen in Liberia, 2021–2024. Monrovia, Liberia: Liberia Ministry of Health (2021).

[2] Kitutu FE Rahman AE Graham H King C El Arifeen S Ssengooba F . Announcing the lancet Global Health Commission on medical oxygen security. Lancet Glob Health. (2022) 10:E1551–2. doi: 10.1016/S2214-109X(22)00407-7, PMID: 36162427

[3] Ross M Wendel SK. Oxygen inequity in the COVID-19 pandemic and beyond. Glob Health Sci Pract. (2023) 11:e2200360. doi: 10.9745/GHSP-D-22-00360, PMID: 36853634 PMC9972372

[4] Bondoe C Wraynee AB Riner ME Allam E Stephenson E. Helping babies breathe: providing an evidence-based education intervention at a tertiary referral hospital in Liberia. J Nurs Educ Pract. (2014) 4:119–27. doi: 10.5430/jnep.v4n9p119

[5] Chang MP Walters CB Tsai C Aksamit D Kateh F Sampson J. Evaluation of fa neonatal resuscitation curriculum in Liberia. Children. (2019) 6:56. doi: 10.3390/children6040056, PMID: 30965659 PMC6517966

[6] Liberia Ministry of Health. Liberia every newborn action plan: strategy and actions to reduce preventable newborn deaths and stillbirths, 2019–2023. Monrovia, Liberia: Liberia Ministry of Health (2019).

[7] Liberia Ministry of Health and Clinton Health Access Initiative. Liberia oxygen assessment report. Monrovia, Liberia: Liberia Ministry of Health (2021).

[8] Liberia Ministry of Health. National medical oxygen quantification report (UNICEF OSPT tool). Liberia: Liberia Ministry of Health (2023).

[9] Nantanda R Kayingo G Jones R van Gemert F Kirenga BJ. Training needs for Ugandan primary care health workers in management of respiratory diseases: a cross-sectional survey. BMC Health Serv Res. (2020) 20:402. doi: 10.1186/s12913-020-05135-3, PMID: 32393227 PMC7212561

[10] Ajeani J Ayiasi RM Tetui M Ekirapa-Kiracho E Namazzi G Kananura RM . A cascade model of mentorship for frontline health workers in rural health facilities in eastern Uganda: processes, achievements and lessons. Glob Health Action. (2017) 10:1345497. doi: 10.1080/16549716.2017.1345497, PMID: 28816629 PMC5645691

[11] Manzi A Hirschhorn LR Sherr K Chirwa C Baynes C Awoonor-Williams JK . Mentorship and coaching to support strengthening healthcare systems: lessons learned across the five population health implementation and training partnership projects in sub-Saharan Africa. BMC Health Serv Res. (2017) 17:831. doi: 10.1186/s12913-017-2656-7, PMID: 29297323 PMC5763487

[12] World Health Organization. (2006). Planning consultation on clinical mentoring approaches and tools to support scaling-up of antiretroviral therapy and HIV care in low-resource settings, Geneva, Switzerland, 7–8 March 2005: working meeting on clinical mentoring approaches and tools to support the scaling-up of antiretroviral therapy and HIV care in low-resource settings, Kampala, Uganda, 16–18 June 2005. Available at: https://iris.who.int/bitstream/handle/10665/43555/9789241594684_eng.pdf?sequence=1&isAllowed=y (Accessed September 1, 2024).

[13] Dauncey JW Olupot-Olupot P Maitland K. Healthcare provider knowledge and practices relating to acute oxygen therapy in Ugandan hospitals: a cross-sectional survey. PLoS One. (2019) 14:e0214122. doi: 10.1371/journal.pone.0214122, PMID: 30913280 PMC6435171

[14] Bakare AA Graham H Ayede AI Peel D Olatinwo O Oyewole OB . Providing oxygen to children and newborns: a multi-faceted technical and clinical assessment of oxygen access and oxygen use in secondary-level hospitals in Southwest Nigeria. Int Health. (2020) 12:60–8. doi: 10.1093/inthealth/ihz009, PMID: 30916340 PMC6964224

[15] Rowe AK Rowe SY Peters DH Holloway KA Chalker J Ross-Degnan D. Effectiveness of strategies to improve health-care provider practices in low-income and middle-income countries: a systematic review. Lancet Glob Health. (2018) 6:E1163–75. doi: 10.1016/S2214-109X(18)30398-X, PMID: 30309799 PMC6185992

